# Reconsideration of the resection strategy of eloquent brain metastasis in the era of postoperative stereotactic radiotherapy: a comparative analysis with non-eloquent metastasis

**DOI:** 10.1007/s11060-025-05075-0

**Published:** 2025-05-23

**Authors:** Levin Häni, Danial Nasiri, Antonia Gächter, Artem Klimov, Mattia Branca, Nicole Söll, Andreas Raabe, Daniel M. Aebersold, Evelyn Herrmann, Ekin Ermiş, Sonja Vulcu, Nicolas Bachmann, Philippe Schucht

**Affiliations:** 1 Department of Neurosurgery, Inselspital, Bern University Hospital, University of Bern, Freiburgstrasse, Bern, Switzerland; 2 Department of Radiation Oncology, Inselspital, Bern University Hospital, University of Bern, Bern, Switzerland; 3 CTU Bern, University of Bern, Bern, Switzerland

**Keywords:** Brain metastasis, Resection, Stereotactic radiotherapy, Radiosurgery, Surgical margin, Eloquence

## Abstract

**Purpose:**

To decrease the recurrence rate after complete resection of a brain metastasis, removal of a surgical safety margin is advocated. This is not always feasible when resecting a metastasis in an eloquent location. We aimed to assess the recurrence rate after resection of metastases in an eloquent location followed by postoperative stereotactic radiotherapy to the resection cavity.

**Methods:**

We retrospectively included patients with 1–3 brain metastases undergoing gross total resection and postoperative stereotactic radiotherapy between 2010 and 2022. Primary endpoint was local recurrence free survival (LRFS). Secondary endpoints were overall survival and distant brain failure free survival. Patients were grouped according to the location of their metastasis into eloquent and non-eloquent. Eloquent localization was considered a surrogate for resection without a surgical safety margin according to our institutional practice.

**Results:**

We included 193 patients with 201 resected metastases. Ninety-five metastases (47.3%) were classified as eloquent and 106 (52.7%) as non-eloquent. Kaplan–Meier analysis showed no difference in LRFS between eloquent and non-eloquent metastases (HR 0.821, 95%-CI 0.447–1.507, p = 0.523). Only increased preoperative tumor volume was associated with worse LRFS (HR 1.015, 95% CI 1.001–1.028, p = 0.033). There was no difference concerning secondary endpoints between eloquent and non-eloquent metastases.

**Conclusion:**

Omission of a surgical safety margin in at least a part of the resection cavity due to eloquence of adjacent tissue had no detrimental effect on local control after resection and postoperative stereotactic radiotherapy of a brain metastasis. This could influence the strategy during resection of an eloquent metastasis.

**Supplementary Information:**

The online version contains supplementary material available at 10.1007/s11060-025-05075-0.

## Introduction

Brain metastases are the most frequent central nervous system tumors and develop in up to 25% of patients with a metastatic malignancy [1]. Previously, surgical resection followed by whole brain radiation therapy (WBRT) represented the standard of care for patients with 1 to 3 metastases [2, 3]. Surgical resection followed by WBRT results in better survival and functional independence, and fewer recurrences compared to WBRT alone [2, 4]. Even though brain metastases are regarded as growing in a well-defined fashion, infiltrative growth beyond the main tumor mass has been reported [5–7]. Consequently, recurrences after complete resection occur in up to 57% of patients after 1 year if no adjuvant radiation is administered [8]. To decrease the recurrence rates, many surgeons advocate the removal of an additional safety margin extending approximately 3–5 mm into normal-appearing brain [9, 10].

The introduction of stereotactic radiosurgery (SRS) in the treatment of patients with brain metastases added a powerful tool to the armamentarium of the neuro-oncologist. For resection of a metastasis with a resection cavity of less than 4–5 cm in maximal diameter, single fraction SRS or postoperative stereotactic fractionated radiotherapy (SFRT) to the resection cavity has replaced WBRT [8, 11–13]. Postoperative SRS/SFRT combines the advantage of better local control than observation, and less cognitive side effects than WBRT, while survival is equal to that following WBRT [8, 13–15].

When postoperative SRS/SFRT is administered, a 2 mm margin is typically added around the resection cavity and residual gross tumor volume (GTV) to define the planning target volume (PTV) [16–19]. When SRS/SFRT to the surgical cavity is used instead of postoperative WBRT, the practice of resecting a safety margin around a brain metastasis needs to be reevaluated. Moreover, the resection of a safety margin is not feasible when resecting a metastasis in an eloquent location.

The aim of our study was to compare the recurrence rate of metastases in eloquent and non-eloquent regions after resection followed by postoperative SRS/SFRT to the resection cavity. Because the surgical safety margin is omitted at least partially when operating on eloquent metastases, eloquent location was considered a surrogate for resection with an incomplete safety margin. We hypothesized that recurrence rates for metastases in eloquent and non-eloquent regions would be similar, since postoperative SRS/SFRT encompasses the potential infiltration zone in both cases. Data on these recurrence rates could influence the surgeon’s strategy during resection of eloquent metastases.

## Methods

### Standard protocol approvals, registrations, and patient consents

We conducted a retrospective, single-center, observational study. Approval for this study was granted by the local ethics committee of the canton of Bern, Switzerland (2023–00020). This work is in accordance with the Declaration of Helsinki in its most recent version.

### Patient population

We included consecutive patients with brain metastases treated at our institution between 01.01.2010 and 31.12.2022. Inclusion criteria were (i) 1 to 3 brain metastases, of which at least 1 was treated surgically, (ii) gross total resection (GTR) confirmed by postoperative contrast-enhanced MRI, (iii) administration of postoperative SRS/SFRT to the surgical cavity, (iv) administration of SRS/SFRT to unresected brain metastases (up to 2), (v) age ≥ 18 years, and (vi) Karnofsky performance status (KPS) ≥ 70.

We excluded patients who (i) underwent biopsy only or subtotal resection, (ii) had no SRS/SFRT administered postoperatively, (iii) had radiotherapy delivered in > 10 fractions, (iv) had a history of prior radiotherapy to the head, (v) had no imaging and clinical follow-up data available, or (vi) refused general consent for scientific use of their health-related data.

### Radiation therapy

Single-fraction SRS or hypofractionated postoperative SFRT was administered to the surgical cavity either with a robotic (CyberKnife, Accuray, Sunnyvale, California) or a conventional linear accelerator system (Novalis, BrainLAB, Munich, Germany). In general, patients with a PTV < 15 ml underwent single session SRS, whereas patients with a PTV ≥ 15 ml underwent hypofractionated SFRT in three, five, six or ten sessions. For treatments delivered with the Novalis system, the dose was prescribed to the 80% isodose line. For CyberKnife treatments, the dose was typically prescribed to the 60–80% isodose line. To facilitate comparison between treatment regimens, the biologically effective dose (BED) was calculated for each cavity assuming a tumor α/β ratio of 10. Target delineation and dose prescription followed international consensus guidelines [13, 19], and the radiotherapy procedure was conducted as previously described by Bachmann et al. [20]. Briefly, target volumes were delineated using post-contrast, thin-slice (1 mm) gadolinium-enhanced T1-weighted and T2-weighted MRI sequences, fused with planning CT scans acquired at a 0.75 mm slice thickness. The GTV was defined as the resection cavity, including the surgical tract and any contrast-enhancing areas surrounding the cavity, as well as the adjacent dura. A 2 mm margin was added to the GTV to create the PTV.

### Data analysis

The primary outcome of our analysis was local recurrence free survival (LRFS). Local recurrence was defined as appearance of a new, nodular contrast enhancement in the surgical bed. In case of equivocal imaging findings, follow-up scans including advanced imaging were used to distinguish between recurrence and radiation necrosis. Secondary outcomes were overall survival and distant brain failure free survival. Distant brain failure was defined as the appearance of a new, non-continuous lesion on MRI or CT of the head, or at least a 20% increase in the longest diameter relative to the nadir of an existing, unresected lesion [21]. Patients underwent regular clinical and imaging follow-up at 3-month intervals. If the last available follow-up imaging demonstrated no tumor growth, data were censored at this time point.

Clinical data were extracted from the institute’s electronic patient data management system. We collected information on patients’ sex, age, KPS, neurological deficits at discharge, primary histology, location and number of brain metastases as well as number of fractions, single dose, total dose, BED, prescription isodose and PTV size. Radiotherapeutic data were gathered from treatment planning systems, ensuring a detailed assessment of dosimetric parameters and treatment protocols.

**Fig. 1:**
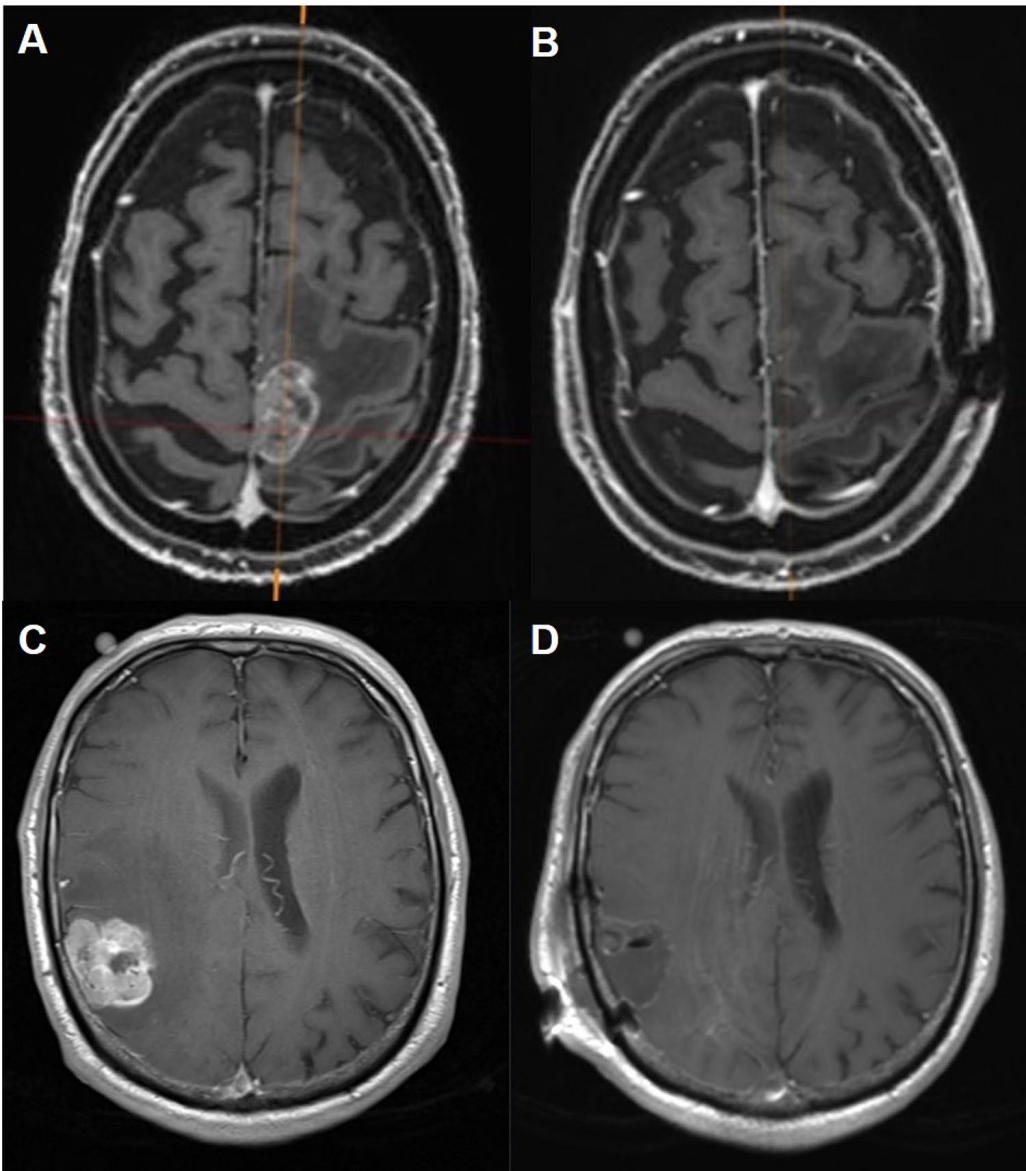
Resection of eloquent and non-eloquent metastases. Axial T1-weighted MR images with contrast are displayed with eloquent (**A** and **B**) and non-eloquent (**C** and **D**) metastasis. Eloquent metastases (**A**) were resected without a safety margin, resulting in a proportionally smaller postoperative resection cavity (**B**). In contrast, non-eloquent metastases (**C**) were resected with an additional safety margin, resulting in a larger resection cavity (**D**)

According to institutional guidelines, metastases in non-eloquent locations were resected with a safety margin of up to 3–5 mm. In contrast, patients with tumors in eloquent locations were resected typically with an incomplete safety margin in at least in a part of the resection cavity, as per our institutional practice (Fig. 1). We considered the following brain areas as eloquent: primary sensory cortex, primary motor cortex, language area, visual cortex, as well as brainstem, hypothalamus, thalamus, internal capsule, cerebellar peduncle, and deep cerebellar nuclei. Metastases in or abutting these regions were considered eloquent. For an additional sensitivity analysis, we compared highly motor eloquent metastases against non-eloquent metastases. A highly motor eloquent location was defined as a metastasis partially or completely in the precentral gyrus.

### Statistics

Statistical analysis was performed using the statistical software SPSS (IBM, Version 28.0). Descriptive statistics included calculation of the mean and standard deviation for normally distributed data as well as the median and interquartile range with lower and upper quartile (IQR) for skewed data. Normal distribution was assessed graphically using Q–Q plots and boxplots as well as analytically using the Shapiro–Wilk test.

For two-way comparison of continuous data, we used a Mann–Whitney U-test. Comparison between groups for nominal variables was made using a chi-square test or Fisher’s exact test.

Kaplan–Meier analysis was used to assess the association between outcome parameters and the resection status, patient characteristics, and tumor characteristics. Differences between groups were examined using the log-rank test for categorical variables, and univariable Cox regression for continuous variables. For multivariable analysis, a Cox regression was conducted including variables based on purposeful selection and a univariable p-value threshold of 0.20.

We addressed missing values first by re-analyzing the source data or, if no value was retrievable, by pairwise deletion.

### Data availability

The study data are available and will be shared upon reasonable request from other investigators for the purposes of replicating results.

## Results

### Patient population

**Fig. 2:**
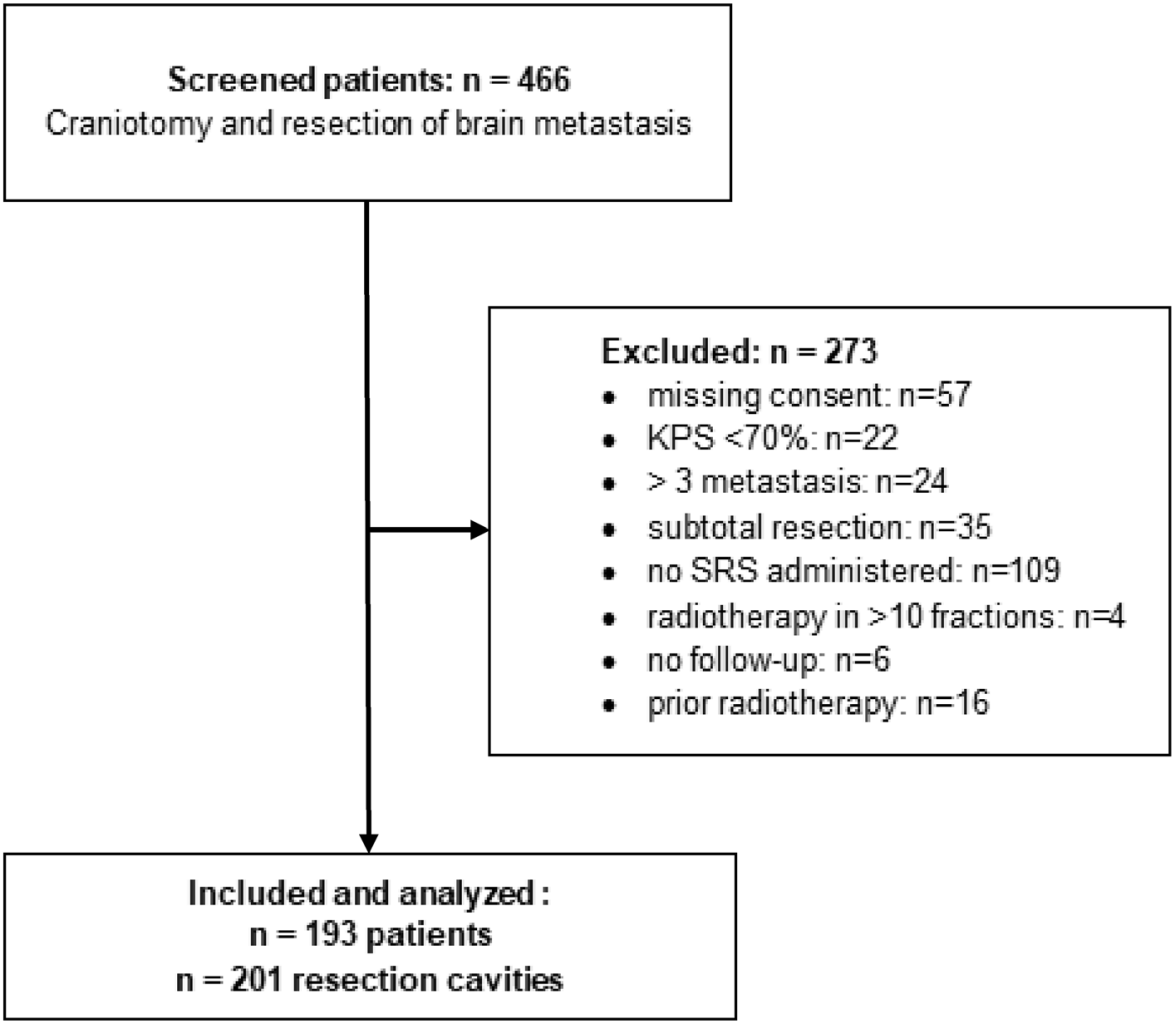
Study flow chart

During the study period, 466 patients underwent resection for brain metastases. After exclusions, the final study population comprised 193 patients with 201 resection cavities (Fig. 2). Median age was 64.0 years (IQR 55.0–72.1). Ninety-three patients (48.2%) were female. Origin of the primary tumor was lung in 94 (48.7%), melanoma in 27 (14.0%), breast in 19 (9.8%), colorectal in 17 (8.8%), renal cell carcinoma in 7 (3.6%), and other in 29 patients (15.0%). Median follow-up duration was 19.4 months (IQR 9.8–35.3).

Postoperative SRS in a single fraction was applied to 85 resection cavities with a median dose of 18 Gy (IQR 15.0–18.0) and a median BED of 50.4 Gy (IQR 37.5–50.4). SFRT in multiple fractions was applied to 116 resections cavities with a median single dose of 6.0 Gy (IQR 6.0–6.0), a median total dose of 30.0 Gy (IQR 27.0–30.0) and a median BED of 48.0 Gy (IQR 43.7–48.0). Among patients treated by postoperative SFRT, 3 fractions were applied to 25 (21.6%), 5 fractions to 79 (68.1%), 6 fractions to 6 (5.2%) and 10 fractions to 6 (5.2%) resection cavities.

Of the 201 resected metastases, 95 were classified as eloquent (47.3%) and 106 as non-eloquent (52.7%). The sites of eloquence were as follows: motor (n = 36), visual (n = 26), sensory (n = 15), language (n = 14), deep cerebellar nuclei (n = 2), thalamus (n = 1) and internal capsule (n = 1). Except for the more frequent occurrence of neurological deficits with eloquent metastases (84.2% versus 36.8%; p < 0.001), eloquent and non-eloquent metastases demonstrated similar characteristics (Table 1). Among patients with a neurological deficit, neurological recovery within 30 days was observed in 69.7% of patients with non-eloquent metastases, compared to 50.0% of those with eloquent metastases (p = 0.058). Statistically, there was a difference in the prescription isodose between eloquent and non-eloquent metastases resulting from more outliers in the former group. This results from the more frequent use of the robotic linear accelerator system in the former group, where the dose was typically prescribed to the 60–80% isodose line in contrast to a fix prescription to the 80% isodose line with the conventional linear accelerator system.

**Table 1:** Baseline characteristics of non-eloquent and eloquent metastases

Variable	Non-eloquent metastases (n = 106)	Eloquent metastases (n = 95)	p-value
Sex (female)	50 (47.2%)	47 (49.5%)	0.744
Age (years)	64.2 (53.1–73.0)	62.0 (54.0–70.0)	0.422
Primary histology - lung - melanoma - breast - colorectal - renal - other	52 (49.1%) 15 (14.2%) 10 (9.4%) 10 (9.4%) 3 (2.8%) 16 (15.1%)	47 (49.5%) 13 (13.7%) 9 (9.5%) 8 (8.4%) 4 (4.2%) 14 (14.7%)	0.997
Preoperative tumor volume (ml)	12.7 (4.7–21.6)	10.4 (3.9–25.3)	0.766
Radiotherapy - Single fraction SRS - SFRT (3 fractions) - SFRT (5 fractions) - SFRT (6 fractions) - SFRT (10 fractions)	44 (41.5%) 14 (13.2%) 41 (38.7%) 5 (4.7%) 2 (1.9%)	41 (43.2%) 11 (11.6%) 38 (40.0%) 1 (3.0%) 4 (4.2%)	0.506
Prescription isodose (%)	80.0 (76.15–80.0)	79.0 (72.0–80.0)	0.015
Robotic (vs conventional) LINAC	76 (71.7%)	82 (86.3%)	0.012
BED - < 50 Gy - ≥ 50 Gy	67 (63.2%) 39 (36.8%)	68 (71.6%) 27 (28.4%)	0.207
PTV (ml)	13.7 (8.8–24.4)	12.5 (8.0–24.8)	0.477
Neurological deficit	39 (36.8%)	80 (84.2%)	< 0.001

Baseline characteristics among eloquent and non-eloquent metastases are displayed by median and IQR for continuous variables as well as absolute number and percentage for nominal variables

*BED* biologically effective dose, *LINAC* linear accelerator system, *SRS* stereotactic radiosurgery, *SFRT* stereotactic fractionated radiotherapy, *PTV* planning target volume

### Local recurrence free survival (LRFS)

**Fig. 3:**
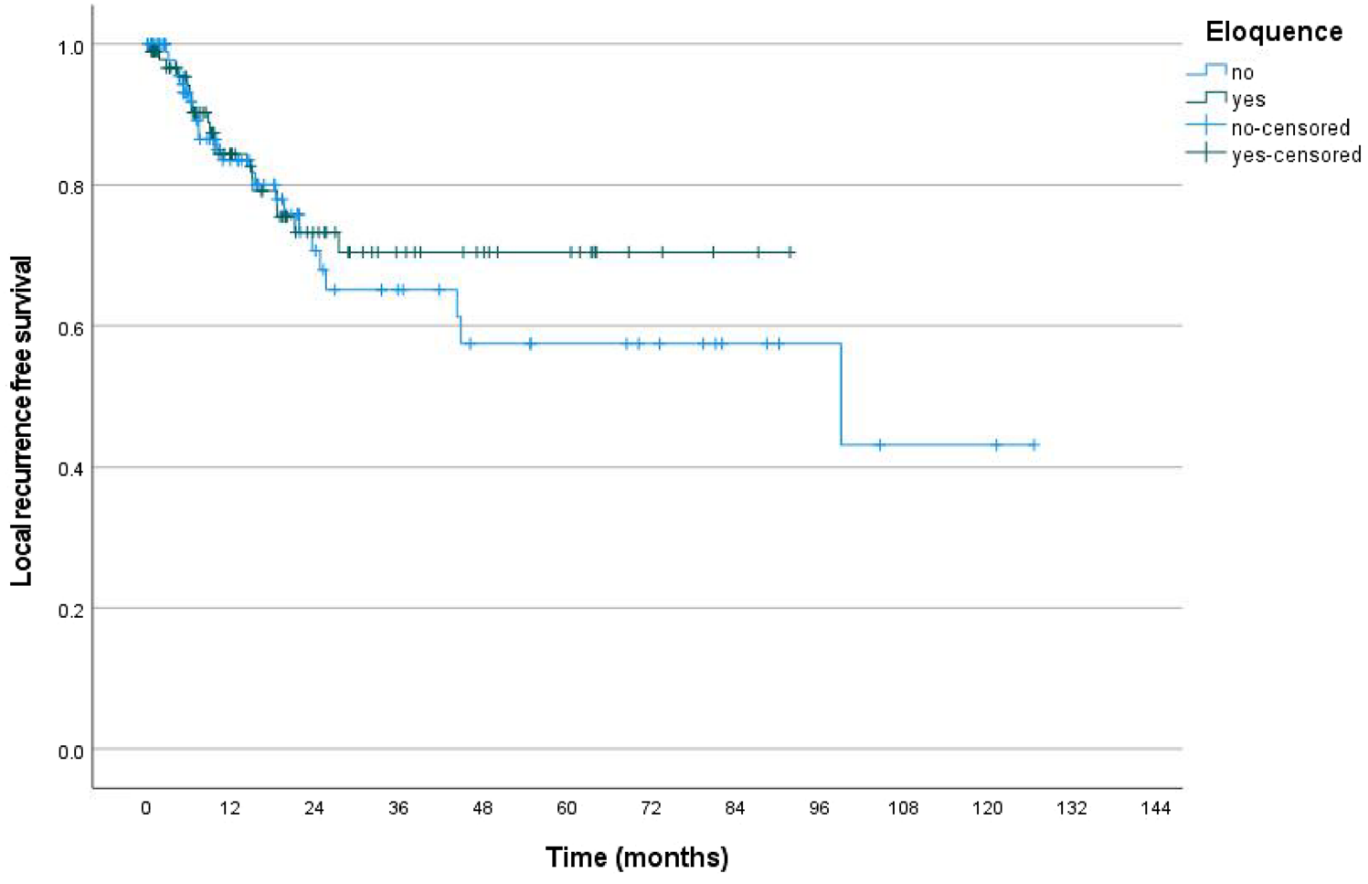
Kaplan–Meier analysis of the primary outcome. Kaplan–Meier analysis of local recurrence free survival found no difference between eloquent and non-eloquent metastases after resection and postoperative SRS/SFRT (p = 0.523)

Local recurrence occurred in 19/95 (20%) of eloquent metastases and 24/106 (22.6%) of non-eloquent metastases (p = 0.684). Among locally recurrent tumors, median time to local recurrence was 9.6 months (IQR 5.7–18.5) in the entire cohort. Kaplan–Meier analysis showed no difference in LRFS when comparing eloquent and non-eloquent metastases (HR 0.821, 95%-CI 0.447–1.507, p = 0.523, Fig. 3). Moreover, there was no difference between individual eloquent areas and non-eloquent metastases (p = 0.910). The 1-year local control rate was 84.4% for eloquent and 83.5% for non-eloquent metastases. The 2-year local control rate was 73.3% for eloquent and 70.7% for non-eloquent metastases.

Results of uni- and multivariable analysis of the association between variables and LRFS are given in Table 2. In the univariable model, only increased preoperative tumor volume was associated with worse LRFS (HR 1.015, 95% CI 1.001–1.028, p = 0.033). In a multivariable model, increased preoperative tumor volume retained its association with a worse LRFS (HR 1.019, 95% CI 1.004–1.034, p = 0.014) (Table 2).

**Table 2:** Uni- and multivariable analysis of the association between variables and local recurrence free survival

Variable	Univariable HR (95%-CI)	p-value	Multivariable HR (95%-CI)	p-value
Sex (female)	1.208 (0.663–2.200)	0.538		
Age	0.995 (0.969–1.020)	0.678		
Primary histology		0.515		
Preoperative tumor volume	1.015 (1.001–1.028)	0.033	1.019 (1.004–1.034)	0.014
Hypofractionated SFRT (vs single fraction SRS)	0.627 (0.341–1.152)	0.133	0.591 (0.253–1.379)	0.224
Prescription isodose (%)	1.006 (0.994–1.143)	0.072	1.049 (0.978–1.124)	0.181
BED < 50 Gy (vs BED ≥ 50 Gy)	0.635 (0.347–1.162)	0.141	0.795 (0.364–1.737)	0.565
PTV (ml)	0.997 (0.974–1.022)	0.835		
Neurological deficit	0.574 (0.312–1.054)	0.073	0.815 (0.354–1.876)	0.631
Eloquence	0.821 (0.447–1.507)	0.523	1.030 (0.486–2.184)	0.939

On univariable analysis, only larger preoperative tumor volume was associated with a decreased local recurrence free survival. On multivariable analysis, none of the variables was associated with local recurrence free survival

*BED* biologically effective dose, *SRS* stereotactic radiosurgery, *SFRT* stereotactic fractionated radiotherapy, *PTV* planning target volume

A sensitivity analysis comparing only highly motor eloquent metastases (n = 20) against non-eloquent metastases (n = 106) estimated no difference in recurrence rate (25% vs 22.6%, p = 0.779) and LRFS (HR 1.055, 95% CI 0.399–2.791, p = 0.913).

### Overall survival

Among carriers of eloquent metastases, 61.1% died during follow-up, whereas 62.3% of those with non-eloquent metastases did so (p = 0.860). Median overall survival was 19.4 months (IQR 9.8–35.3) in the entire cohort. Kaplan–Meier analysis showed no difference in overall survival between patients with eloquent and non-eloquent metastases (HR 0.930, 95%-CI 0.651–1.327, p = 0.688, supplementary Fig. 1). One-year survival rate was 71.7% in patients with non-eloquent and 74.4% in patients with eloquent metastases.

On uni- and multivariable analysis, increasing age was associated with significantly worse overall survival (multivariable HR 1.024, 95% CI 1.006–1.043, p = 0.008). In addition, there was a significant association of survival with primary histology (multivariable p = 0.014). Survival was worst for colorectal cancer and the category of various other primary tumors, whereas it was best for melanoma and renal cell carcinoma. No other association with survival was found on uni- and multivariable analysis (supplementary Table 1).

### Distant brain failure

Distant brain failure occurred in 38.9% of hosts of eloquent metastases and 42.5% of those with non-eloquent metastases (p = 0.614). Kaplan–Meier analysis showed no difference in distant brain failure free survival between patients with eloquent and non-eloquent metastases (HR 0.855, 95% CI 0.549–1.330, p = 0.487). Among patients suffering from distant brain failure, median time to distant brain failure was 6.6 months (IQR 4.3–12.6). None of the variables showed an association with distant brain failure free survival.

Leptomeningeal disease developed in 4.2% of eloquent tumors and 3.8% of non-eloquent tumors (p = 0.577).

## Discussion

Our results show no differences in the local control rates, irrespective of eloquent or non-eloquent location, when postoperative SRS/SFRT is delivered to the resection cavity. In contrast, increased preoperative tumor volume was associated with worse local control. Importantly, tumor volume might also exert its influence on local control through radiosurgical constraints in larger lesions.

### Do we need a safety margin for an eloquent metastasis?

Invasion of brain metastases into surrounding parenchyma with a distance from the main tumor mass of up to 0.450 mm was reported by Berghoff et al. [5]. Baumert et al. reported a maximal depth of infiltration of up to 1–2 mm among non-small cell lung cancer and melanoma metastases [6]. Yoo et al. observed a dramatic improvement of local control, with a decrease in the 1-year local recurrence rate from 58.6% to 29.1%, when an additional safety margin of 5 mm was resected around a brain metastasis [22]. However, postoperative radiotherapy was performed in only 30.9% of patients in that series and the effect of resecting a safety margin might therefore have been overestimated. Some brain metastases occur in eloquent areas where removal of a safety margin might not be possible. Now that postoperative SRS/SFRT to the resection cavity is widely used, the value of a safety margin should be reevaluated and balanced against the risk of inducing a neurological deficit. Postoperative SRS/SFRT is likely to cover the infiltration zone, because a 1–2 mm margin around the resection cavity is added to define the PTV [16–18]. Supporting this hypothesis, our results indicate that the omission of a safety margin is not detrimental to local control when using eloquence as a surrogate for an incomplete surgical safety margin in at least a part of the resection cavity. This raises the question of whether a safety margin is necessary in all brain metastases, irrespective of location. While a more extensive resection may theoretically improve local control, it also increases the irradiated volume, potentially leading to a higher risk of radiation-induced toxicity. This is particularly relevant for lesions in non-eloquent areas, where more aggressive resections are often considered feasible. Therefore, our findings suggest that the need for a surgical safety margin should be carefully weighed against the risk of postoperative complications, especially in the context of modern radiotherapy techniques.

The importance of the extent of resection of brain metastases has recently been questioned. Jünger et al. found no influence of the extent of resection on the local recurrence rate and overall survival in a contemporary series of 197 patients with brain metastases [23]. In contrast, before the introduction of postoperative SRS, Agboola et al. reported a significantly better survival in patients with completely resected brain metastases compared to incomplete resection in their series [24]. Likewise, Lee et al. reported better survival after GTR compared to subtotal resection (STR) in a retrospective series of 157 cases [25]. However, the local recurrence rate did not differ between patients undergoing STR and GTR, raising the question whether overall survival was influenced by systemic factors rather than by the extent of resection.

### Clinical implications

It seemed that the completeness of the safety margin had no or only a minor effect on local control. This finding may influence the surgical strategy when resecting eloquent lesions. A surgically induced new neurological deficit affects quality of life and is associated with worse survival and must therefore be avoided [26]. If functional eloquence prohibits the resection of an additional safety margin, surgeons might adjust their strategy and leave residual microscopic disease to postoperative SRS/SFRT.

In our cohort, local control did not differ between postoperative single-fraction SRS and hypofractionated SFRT. Nevertheless, increased preoperative tumor volume was associated with worse local control. This is in line with the results of Hartford et al. who reported a shorter time to recurrence and to salvage WBRT after resection and postoperative SRS of larger metastases [27]. Hypofractionated SFRS in 3 to 5 sessions is often applied for surgical cavities ≥ 15 ml. Assuming a spherical shape, this translates into a sphere diameter of 3 cm, which is already smaller than many surgical lesions. Thus, particularly for large metastases in non-eloquent brain areas, the resection of a safety margin might still be appropriate when anticipating radiosurgical constraints due to the tumor size. On the other hand as discussed above, the use of a surgical safety margin increases the volume to be irradiated, potentially leading to a higher risk of radiation-induced complications, such as radiation necrosis. While our study did not specifically analyze radiation necrosis rates, existing literature suggests that GTV to PTV expansions of 0 to 3 mm achieve comparable 1-year local tumor control rates without a significantly increased risk of radiation necrosis [28–30]. Considering the impact of surgical safety margins on postoperative irradiation volume, a reduced or even margin-free GTV/PTV expansion strategy could be explored. However, further research is necessary to validate the safety and efficacy of such an approach.

Suki et al. reported an increased incidence of leptomeningeal dissemination after piecemeal resection compared to en bloc resection [31]. Thus, concerns might arise because a resection without a safety margin can be considered piecemeal per se. A detailed analysis of piecemeal versus en bloc resection was not feasible in our cohort. Nevertheless, leptomeningeal disease was a rare occurrence and did not differ between the eloquent and non-eloquent group.

Interestingly, the defense of glial cells against foreign metastatic cells during colonization is organ-specific [6, 7]. Depending on the primary histology, some brain metastases show predominantly infiltrative growth, while others do not [6, 7]. This is beyond the scope of our work and further studies are needed, but the decision to resect a safety margin might also be influenced by the primary histology and adjuvant oncological treatment options.

### Limitations

Several limitations apply to our study. Our results were obtained by a retrospective analysis of data from a single center. Furthermore, the cohort size is limited and the study might be underpowered. In addition, our cohort comprised patients with metastases that had different primary tumors with different tissue textures, consistencies, biological aggressiveness and prognosis.

We used eloquent location as a surrogate for a higher likelihood of an incomplete surgical safety margin, since the dogma of maximum, but safe resection reflects the contemporary neuro-oncological practice. However, a surgeon might still have removed a safety margin around an eloquent metastasis based on the neurophysiological findings without detailing this in the operative report. Thus, we cannot exclude possible misclassification, which could have led to the difference between groups becoming blurred. In addition, a certain interobserver variability is inevitable, since different surgeons might follow different strategies depending on the location of the lesion. Consequently, the scientific validity of eloquence as a surrogate for the resection strategy is unclear, which limits generalizability of our results.

Moreover, classifying follow-up images into true recurrence versus post-therapeutic changes can be difficult and further dilute the reliability of the results.

## Conclusion

In our patients undergoing resection of a brain metastasis, partial omission of the safety margin due to eloquence of the surrounding brain had no detrimental effect on local control, when postoperative SRS/SFRT to the resection cavity was administered. These data could influence the surgeon’s strategy during resection of an eloquent metastasis, prioritizing neurological safety over resection of a safety margin. Prospective studies are needed to confirm our findings.

## Supplementary Information

Below is the link to the electronic supplementary material.

Supplementary file1 (DOCX 65 KB)

## Data Availability

No datasets were generated or analysed during the current study.

## Abbreviations

95%-CI: 95% Confidence interval
BED: Biologically effective dose
GTR: Gross total resection
GTV: Gross tumor volume, HR: hazard ratio
IQR: Interquartile range
KPS: Karnofsky performance status
LINAC: Linear accelerator system
LRFS: Local recurrence free survival
PTV: Planning target volume
SFRT: Stereotactic fractionated radiotherapy
SRS: Stereotactic radiosurgery
STR: Subtotal resection
WBRT: Whole brain radiation therapy
