# Supplementary material for: Reconsideration of the resection strategy of eloquent brain metastasis in the era of postoperative stereotactic radiotherapy: a comparative analysis with non-eloquent metastasis
Source: J Neurooncol. 2025 May 23;174(2):439–48. doi: 10.1007/s11060-025-05075-0 (PMC12208965; doi:10.1007/s11060-025-05075-0)
Supplement: Supplementary file 1 — Supplementary file1 (DOCX 65 KB) [file 11060_2025_5075_MOESM1_ESM.docx]

**Reconsideration of the resection strategy of eloquent brain metastasis in the era of postoperative stereotactic radiotherapy: A comparative analysis with non-eloquent metastasis**

Levin Häni^1^, MD^#^; Danial Nasiri^1^, MD^#^; Antonia Gächter^2^, MD; Artem Klimov^1^, BSc; Mattia Branca^3^, PhD; Nicole Söll^1^; Andreas Raabe^1^, MD; Daniel M Aebersold^2^ MD; Evelyn Herrmann^2^, MD; Ekin Ermiş^2^, MD; Sonja Vulcu^1^, MD; Nicolas Bachmann^2^, MD*; and Philippe Schucht^1^, MD*

^1^Department of Neurosurgery, Inselspital, Bern University Hospital, University of Bern, Bern, Switzerland

^2^Department of Radiation Oncology, Inselspital, Bern University Hospital, University of Bern, Bern, Switzerland

^3^CTU Bern, University of Bern, Bern, Switzerland

^#^ = Levin Häni and Danial Nasiri share the first authorship of this work.

* = Nicolas Bachmann and Philippe Schucht share the senior authorship of this work.

Keywords: *brain metastasis, resection, stereotactic radiotherapy, radiosurgery, surgical margin, eloquence*

**SUPPLEMENTARY APPENDIX**

**SUPPLEMENTAL FIG. 1 Kaplan-Meier analysis of overall survival**


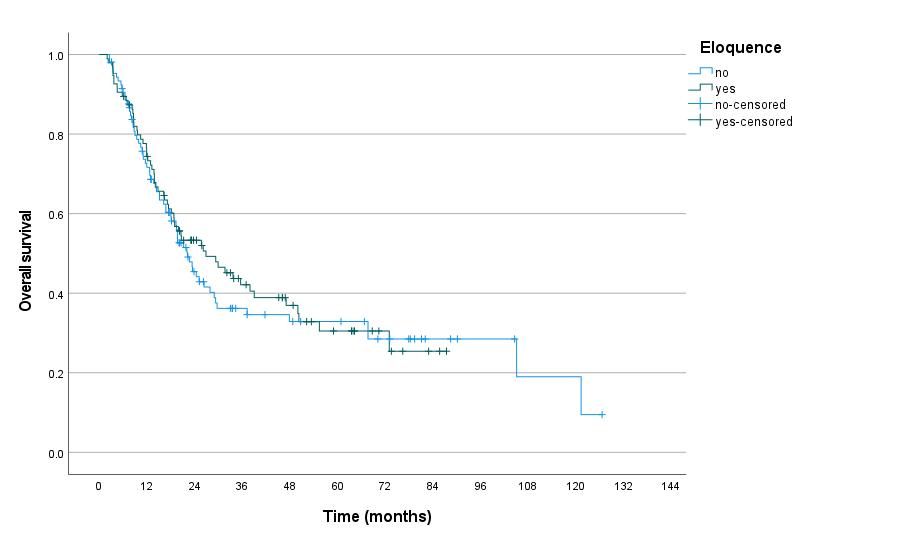


Kaplan-Meier analysis of overall survival did not show a significant difference between hosts of eloquent and non-eloquent metastasis (p=0.688).

**SUPPLEMENTAL TABLE 1. Uni- and multivariable analysis of the association between variables and overall survival.**

| **Variable** | **Univariable**  **HR (95%-CI)** | **p-value** | **Multivariable**  **HR (95%-CI)** | **p-value** |
| --- | --- | --- | --- | --- |
| **Sex (female)** | 1.002 (0.704-1.427) | 0.991 |  |  |
| **Age** | 1.024 (1.008-1.041) | 0.004 | 1.024 (1.006-1.043) | 0.008 |
| **Primary histology** |  | 0.011 |  | 0.014 |
| **Preoperative tumor volume** | 1.007 (0.998-1.016) | 0.120 | 1.005 (0.996-1.014) | 0.260 |
| **Hypofractionated SFRT (vs single fraction SRS)** | 0.983 (0.687-1.406) | 0.925 |  |  |
| **Prescription isodose (%)** | 1.017 (0.985-1.050) | 0.304 |  |  |
| **BED <50 Gy (vs BED ≥50 Gy)** | 1.024 (0.703-1.491) | 0.901 |  |  |
| **PTV (ml)** | 1.011 (0.998-1.023) | 0.092 |  |  |
| **Neurological deficit** | 1.062 (0.739-1.527) | 0.744 |  |  |
| **Eloquence** | 0.930 (0.651-1.327) | 0.688 | 0.844 (0.586-1.215) | 0.361 |

On uni- and multivariable analysis, only increasing age and histology of the primary tumor were associated with worse overall survival. Survival was worst among patients with colorectal cancer and the category of various primaries. BED = biologically effective dose; SRS = stereotactic radiosurgery; SFRT = stereotactic fractionated radiotherapy; PTV = planning target volume.
